# Clinical efficacy of membrane anatomy-based 3D laparoscopic partial nephrectomy in a single stage for the treatment of ruptured renal angiomyolipoma

**DOI:** 10.3389/fonc.2026.1819091

**Published:** 2026-05-29

**Authors:** Jiansheng Xiao, Handa Zheng, Yuxiang Liu, Zhihui Chen, Feng Luo, Guohao Wu, Shaoqiang Ye, Ping Li, Taifen Kang, Huilan Luo, Caiyong Lai, Dongming Ye

**Affiliations:** 1 Department of Urology, The Sixth Affiliated Hospital of Jinan University, Dongguan Eastern Central Hospital, Dongguan, China; 2 Department of Minimally Invasive Interventional Vascular Surgery, The Sixth Affiliated Hospital of Jinan University, Dongguan Eastern Central Hospital, Dongguan, China; 3 Department of Urology, Miyun Hospital, Peking University First Hospital, Beijing, China

**Keywords:** embolization, laparoscopic partial nephrectomy, membrane anatomy, renal angiomyolipoma, total intracorporeal laparoscopy

## Abstract

**Objective:**

This study aims to describe the preliminary single-center clinical experience with membrane anatomy-based 3D laparoscopic partial nephrectomy (LPN) performed in a single stage for selected patients with ruptured renal angiomyolipoma (RAML), and to assess its perioperative feasibility and short-term outcomes.

**Methods:**

A single-center retrospective descriptive design was employed to collect clinical data from 12 patients with ruptured renal angiomyolipoma who were treated between January 2020 and December 2025. All patients underwent 3D LPN via a transabdominal approach, with strict adherence to membrane anatomy principles during surgery.

**Results:**

A total of 12 patients underwent 3D laparoscopic surgery for ruptured renal angiomyolipoma. All patients successfully completed the procedure without conversion to open surgery, and none required radical nephrectomy. The average surgical duration was 311.83 ± 85.46 minutes, with a mean warm ischemia time of 25.5 ± 11.02 minutes. Intraoperative blood loss averaged 327.00 ± 114.00 mL, and 83.33% of patients required intraoperative blood transfusions. Pain levels progressively decreased after surgery, with average VAS scores of 4.00 ± 1.00 at 24 hours and 3.20 ± 0.84 at 48 hours. Renal function markers showed transient fluctuations. The preoperative mean serum creatinine was 78.44 ± 23.97 μmol/L, which increased to 90.68 ± 28.07 μmol/L postoperatively, and then decreased to 85.55 ± 22.84 μmol/L at the 3-month follow-up. No tumor recurrence was observed during the postoperative follow-up period.

**Conclusion:**

Membrane anatomy-based 3D LPN for selected ruptured RAML cases showed acceptable perioperative safety, renal function preservation, and postoperative recovery, but requires validation in larger prospective comparative studies.

## 1 Introduction

Renal angiomyolipoma (RAML) is a benign renal tumor composed of mature adipose tissue, smooth muscle cells, and abnormally proliferating blood vessels. It is one of the most common benign solid tumors of the kidney, with a higher incidence in females, and some patients are also associated with tuberous sclerosis complex (1, 2). Most RAMLs are small and clinically asymptomatic, often discovered incidentally during routine check-ups or imaging studies. As the tumor enlarges, patients may experience symptoms such as lumbar discomfort, abdominal masses, gross or microscopic hematuria, and compression of the surrounding renal parenchyma, which can lead to renal dysfunction (3, 4). The most severe complication of RAML is spontaneous rupture and hemorrhage, which manifests as sudden, severe lumbar and abdominal pain, retroperitoneal hematoma, and, in extreme cases, hemorrhagic shock, potentially life-threatening. Previous studies have identified tumor enlargement and aneurysm formation within the tumor as major risk factors for significantly increased bleeding, with a tumor diameter of 4 cm commonly serving as the threshold for intervention. For lesions with pronounced symptoms or those showing high-risk vascular morphology on imaging, active treatment is recommended (5, 6).

Current treatment strategies for RAML are mainly individualized based on tumor size, symptoms, and bleeding risk. Smaller, asymptomatic lesions can often be monitored, while larger, rapidly growing tumors, or those that have already ruptured or are highly suspected of rupture, require active intervention (2, 7). Common treatment methods include selective renal artery embolization, partial nephrectomy (PN), and radical nephrectomy. Arterial embolization (AE) can effectively control bleeding in a short period, preserving more renal units and thus becoming a crucial approach for managing high-risk or ruptured RAML (8). However, residual tumors frequently remain, with recurrent bleeding and the need for further interventions being common. Additionally, post-embolization fibrosis and granulation tissue formation, resulting from hematoma organization and inflammation, can complicate subsequent surgeries (9, 10). Surgical procedures, on the other hand, can simultaneously control bleeding and excise the tumor, which is essential for achieving long-term cure. However, traditional early open surgeries or conventional laparoscopic procedures often result in significant blood loss and high nephrectomy rates (11, 12). In recent years, with the development of 3D laparoscopy and the concept of membrane anatomy, surgeons can now more precisely navigate natural spaces, such as the renal fascia, to quickly identify and control the renal vasculature, allowing for nephrectomy and reconstruction based on this approach (13). For patients with acute ruptured hemorrhage of RAML, the renal perinephric anatomical layer often remains intact after bleeding, and the tumor itself is soft and easily compressed. With the help of 3D visualization, aspiration, electrocautery, and the strategic increase in trocar ports for enhanced exposure and retraction, it is possible to achieve effective hemostasis and maximize renal unit preservation during a single-stage surgery (14).

Nevertheless, there is still limited research both domestically and internationally regarding the appropriateness of performing single-stage 3D laparoscopic surgery during the same hemorrhagic event for ruptured RAML. The timing and indications for surgery have not yet reached universal consensus (15). This is especially important in the context of previous studies suggesting that “delayed surgery may have lower perioperative risks,” making the safety and efficacy of performing single-stage surgery in the acute phase under 3D laparoscopy even more critical to investigate. Therefore, this study retrospectively analyzes the clinical data of patients at our center who underwent single-stage surgery using 3D laparoscopy as the first surgical intervention for spontaneous ruptured RAML. The study evaluates perioperative parameters, renal unit preservation, and changes in renal function during follow-up, aiming to provide a reference for determining the appropriate surgical timing and decision-making for such patients.

## 2 Materials and methods

**Figure 1:**
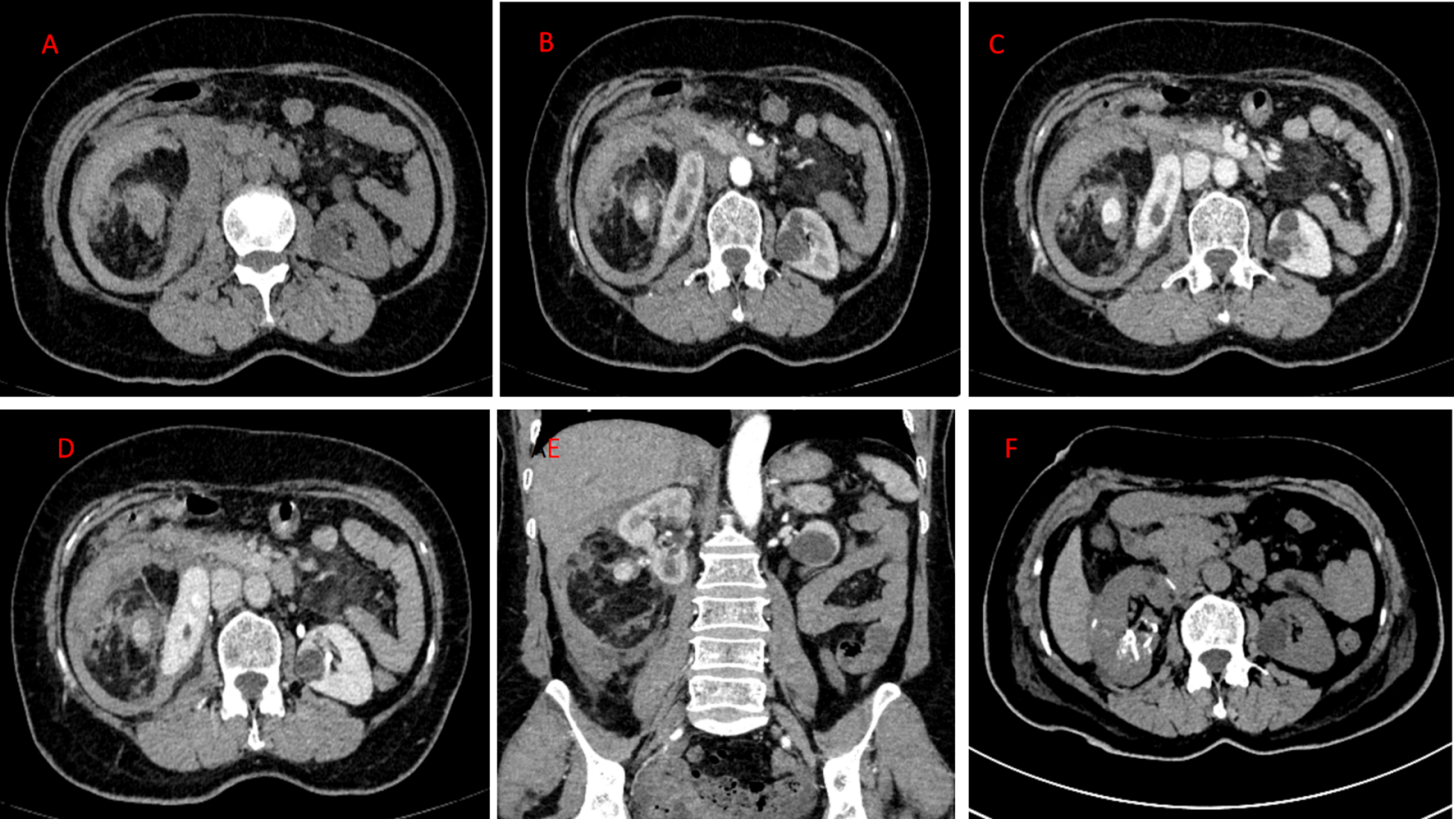
Preoperative and postoperative CT images of the lower abdomen + pelvis with contrast enhancement in patients with renal angiomyolipoma. **(A)** Preoperative non-contrast CT image showing a renal space-occupying lesion; **(B)** Preoperative arterial phase CT image showing uneven enhancement in the lesion area; **(C)** Preoperative venous phase CT image showing changes in the enhancement degree of the lesion over time; **(D)** Preoperative excretory phase CT image displaying the anatomical relationship between the lesion and the renal pelvis and calyces; **(E)** Preoperative coronal reconstruction CT image during the arterial phase, clearly showing the spatial location and extent of the lesion; **(F)** Postoperative follow-up CT image showing good structural recovery of the affected kidney with no significant hematoma or residual lesions.

This study is a single-center, retrospective descriptive case series that includes 12 patients treated with 3D laparoscopy for ruptured RAML at our hospital between January 2020 and December 2025. Ruptured RAML is defined as: the patient presents with gross hematuria, imaging indicates ruptured RAML with bleeding, or intraoperative exploration reveals perirenal or intratumoral hematoma. Preoperative imaging of patients with ruptured renal angiomyolipoma is shown in **Figure 1A**. This study was designed to summarize preliminary clinical experience and short-term perioperative outcomes; it was not designed to compare this strategy with delayed surgery, embolization-only treatment, open surgery, or other nephron-sparing approaches. This study was approved by the Ethics Committee of the Sixth Affiliated Hospital of Jinan University, and all patients signed written informed consent before the procedure, ensuring the compliance and ethical integrity of the study.

**Figure 2:**
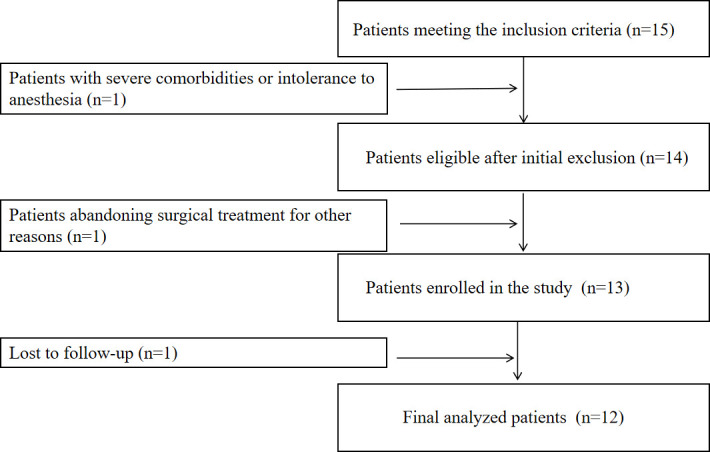
The screening process for this study.

All surgeries in this study were performed by the same urologist, who had more than 10 years of experience in laparoscopic surgery and had received standardized surgical training. To ensure procedural consistency and quality, all operations were conducted according to a standardized surgical protocol. The inclusion and exclusion criteria were strictly applied to minimize selection bias. The inclusion criteria were as follows: 1) ruptured renal angiomyolipoma (RAML) confirmed by imaging examinations, such as computed tomography (CT) or magnetic resonance imaging (MRI); 2) treatment with 3D laparoscopic surgery; 3) age between 18 and 70 years; and 4) complete clinical data, including relevant preoperative, intraoperative, and postoperative parameters. The exclusion criteria were: 1) severe comorbidities or inability to tolerate anesthesia; and 2) refusal of surgery for other reasons. Ultimately, 12 patients who met all eligibility criteria were included in this study, and the patient selection process is shown in **Figure 2**. All procedures were performed using the Storz 3D laparoscopic system, a high-definition system that provides stereoscopic visualization and thereby improves the precision and safety of the surgical procedure.

In this study, all patients first underwent AE to control the ruptured RAML bleeding, ensuring stable blood flow during surgery and minimizing blood loss. Preoperatively, patients routinely received third-generation cephalosporins for anti-infection treatment, and antibiotics were adjusted according to urine culture results when necessary. Patients were given liquid diets two days before surgery and polyethylene glycol electrolytes for bowel cleansing the day before surgery, along with enteral nutrition supplements to ensure intestinal cleanliness. Perioperative management included strict anesthesia monitoring and electrolyte balance during surgery, with postoperative monitoring of blood routine, electrolytes, and renal function within 24 hours to ensure smooth recovery. This combined strategy was intended to stabilize hemorrhage and facilitate subsequent nephron-sparing surgery; however, because no comparator group was included, its relative benefit compared with alternative treatment pathways cannot be determined from the present dataset.

We retrospectively collected demographic, clinical, imaging, intraoperative, and postoperative data from the medical records. Demographic and clinical variables included sex, age, body mass index, major comorbidities, ASA score, presenting symptoms, tumor laterality, tumor location, tumor size, tumor number, cystic degeneration, and surgical difficulty score based on preoperative imaging and clinical symptoms. Perioperative variables included surgical approach, use of arterial embolization, warm ischemia time, operative time, estimated blood loss, transfusion requirement, nephrectomy status, serum creatinine levels, hematocrit, postoperative hospital stay, and postoperative complications. Renal function was assessed using serum creatinine levels before surgery, on the day of surgery, and at 3 months after surgery. Postoperative complications were graded according to the Clavien-Dindo classification system (16). Postoperative pain was evaluated using the Visual Analog Scale (VAS; 0 = no pain, 10 = worst pain) at 24 and 48 hours after surgery by trained staff.

Following surgery for ruptured RAML, patients underwent regular postoperative follow-up. The follow-up protocol included clinical assessment, physical examination, laboratory tests, and imaging surveillance. Laboratory evaluation included serum creatinine, electrolytes, and urine routine tests to monitor renal function and urinary complications. Imaging assessment was performed using abdominal and pelvic CT, with contrast-enhanced CT or MRI when clinically indicated, to evaluate residual lesions, recurrent tumor, recurrent bleeding, perirenal hematoma or fluid collection, and delayed structural changes of the affected kidney. Follow-up visits were scheduled at 1, 3, 6, and 12 months after surgery, and patients were subsequently followed according to their clinical condition. Representative follow-up imaging is shown in **Figure 1**.

### 2.1 Statistical analysis

All statistical analyses were conducted using IBM SPSS Statistics software, version 26.0 for Windows (IBM Corp., Armonk, NY, USA). All numerical data were first tested for normality using the Kolmogorov-Smirnov test. For normally distributed data, the mean ± standard deviation mean ± standard deviation (x̄ ± s) was used, while for non-normally distributed data, the median (P25, P75) was used.

## 3 Results

### 3.1 Demographics and baseline characteristics of patients

A total of 12 patients underwent 3D laparoscopic surgery for ruptured renal angiomyolipoma. The average age of the patients was 54.17 ± 5.92 years, including 7 males and 5 females. The mean BMI was 23.18 ± 3.69 kg/m². According to the ASA classification, 10 patients were classified as ASA II, and 2 patients as ASA III. No patients were classified as ASA I. Two patients had comorbidities, including hypertension and diabetes mellitus. All patients presented with lumbar pain, with hematuria in 2 patients and fever in 4 patients as additional symptoms. Regarding tumor characteristics, all patients had a solitary tumor without cystic degeneration. The tumors were located as follows: 5 in the upper pole, 3 in the middle pole, and 4 in the lower pole. The average tumor size was 6.24 ± 2.07 cm, with 9 tumors smaller than 5 cm and 3 tumors larger than 5 cm. Tumor laterality was balanced, with 6 left-sided and 6 right-sided cases. All patients had no prior renal surgery on the affected side and received preoperative interventional treatment (**Table 1**).

**Table 1:** Demographics and baseline characteristics of patients.

Characteristics	Results
Number of patients, (n)	12
Age (years), mean ± SD	54.17 ± 5.92
Gender, n (%)	
Male	7 (58.3)
Female	5 (41.7)
BMI* (kg/m^2^), mean ± SD	23.18 ± 3.69
ASA *classification, n (%)	
I	0 (0.00)
II	10 (83.33)
III	2 (16.67)
Clinical symptoms, n (%)	
Lumbago	12 (100)
Hematuria	2 (16.67)
Fever	4 (33.33)
Comorbodities	
Hypertension	2 (16.67)
Diabetes mellitus	2 (16.67)
Preoperative hemoglobin (g/L), mean ± SD	99.42 ± 17.10
Preoperative serum creatinine (μmol/L), mean ± SD	78.44 ± 23.97
Characteristics of renal angiomyolipoma	
Location, n (%)	
Upper pole of kidney	5 (41.67)
Middle pole of kidney	3 (25.00)
Lower pole of kidney	4 (33.33)
Tumor size (cm), mean ± SD	6.24 ± 2.07
<5cm	9 (75.00)
>5cm	3 (25.00)
Tumor number, n (%)	
Solitary	12 (100.00)
Multiple	0 (0.00)
Cystic degeneration, n (%)	
Yes	0 (0.00)
No	12 (100.00)
Operated side, n (%)	
Left	6 (50)
Right	6 (50)
Surgical history on the operated side, n (%)	
Yes	1 (8.33)
No	11 (91.67)
Preoperative interventional therapy, n (%)	
Yes	12 (100.00)
No	0 (0.00)

*BMI, Body Mass Index; ASA, American Society of Anesthesiologists.

### 3.2 Intraoperative and postoperative data

**Figure 3:**
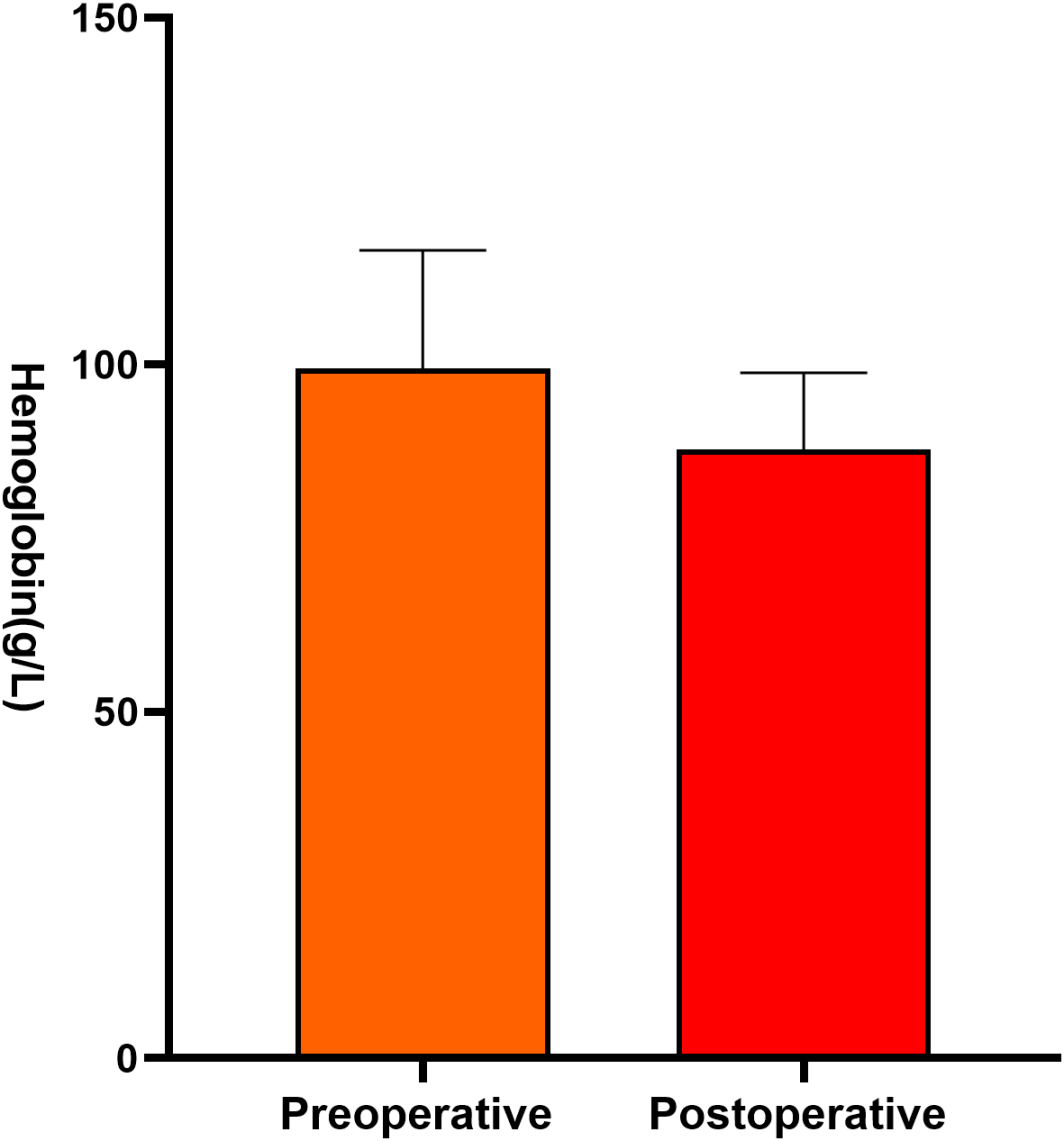
Comparison of preoperative and postoperative hemoglobin.

**Figure 4:**
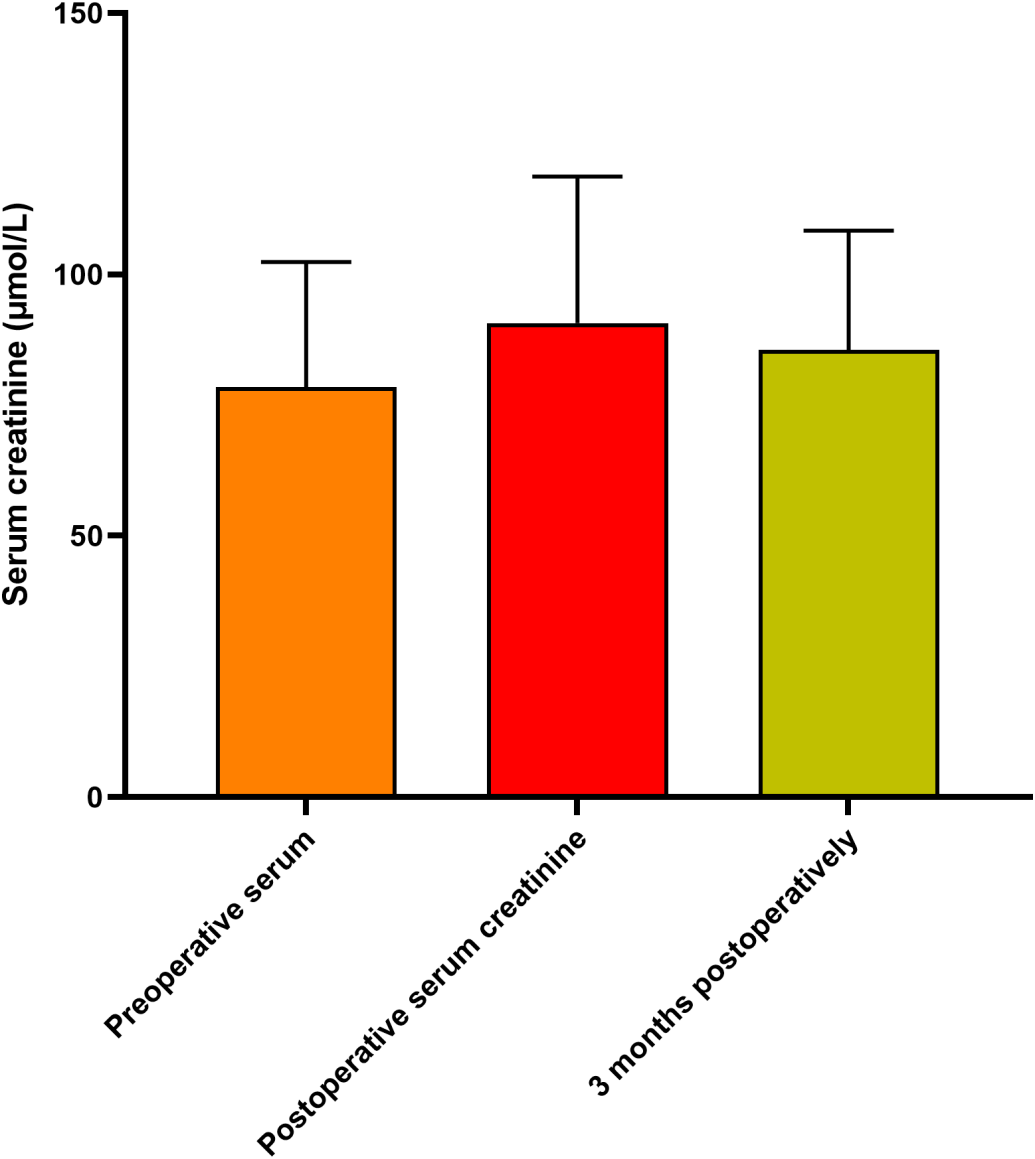
Comparison of serum creatinine levels before surgery, after surgery, and at 3 months postoperative.

All patients successfully underwent transabdominal 3D laparoscopic surgery, with no conversions to open surgery and no radical nephrectomies performed. The average surgical time was 311.83 ± 85.46 minutes, with a mean warm ischemia time of 25.5 ± 11.02 minutes. The average intraoperative blood loss was 327.00 ± 114.00 mL, and 83.33% of patients required intraoperative blood transfusion. The mean postoperative hospital stay was 11.42 ± 2.75 days. Pain scores progressively decreased after surgery, with average VAS scores of 4.00 ± 1.00 at 24 hours and 3.20 ± 0.84 at 48 hours. The preoperative mean hemoglobin was 96.40 ± 22.02 g/L, which decreased to 89.40 ± 13.67 g/L postoperatively (**Figure 3**). Regarding renal function, the preoperative mean serum creatinine was 78.44 ± 23.97 μmol/L, which increased to 90.68 ± 28.07 μmol/L postoperatively, before decreasing to 85.55 ± 22.84 μmol/L at the 3-month follow-up (**Figure 4**). No tumor recurrence was observed during the postoperative follow-up period.

Postoperative complications were evaluated according to the Clavien-Dindo classification. Overall, 2 grade I, 8 grade II, and 3 grade III complication events were recorded. Grade I complications were managed conservatively, whereas grade II complications mainly required pharmacological treatment, blood transfusion, or antibiotic therapy. All three grade III complications were symptomatic or persistent postoperative perirenal fluid collections/hematomas requiring image-guided percutaneous drainage. As these interventions were performed without general anesthesia, they were classified as Clavien-Dindo grade IIIa. All three patients recovered after drainage and supportive treatment without further sequelae. No grade IV–V complications, perioperative death, conversion to radical nephrectomy, or renal unit loss occurred. During a mean follow-up period of 33.25 ± 17.03 months, no tumor recurrence, recurrent bleeding, renal unit loss, or secondary intervention was observed. Serum creatinine showed a transient postoperative increase and decreased at the 3-month follow-up, suggesting generally stable renal function during the available follow-up period (**Table 2**).

**Table 2:** Intraoperative and postoperative data.

Characteristics	Results
Number of patients, n (%)	12
Surgical approach (transabdominal), n (%)	12 (100.00)
Operative time (minutes)	311.83 ± 85.46
Postoperative hospital stays (days), mean ± SD	11.42 ± 2.75
Nephrectomy performed, n (%)	
Yes	0 (0.00)
No	12 (100.00)
Conversion to open surgery, n (%)	
Yes	0 (0.00)
No	12 (100.00)
VAS score	
Postoperative 24 h VAS score, mean ± SD	4.00 ± 1.00
Postoperative 48 h VAS score, mean ± SD	3.20 ± 0.84
Warm ischemia time (minutes), mean ± SD	25.5 ± 11.02
Intraoperative blood loss (mL), mean ± SD	327.00 ± 114.00
Intraoperative blood transfusion rate (%)	10 (83.33)
Postoperative hemoglobin (g/L), mean ± SD	89.40 ± 13.67
Postoperative hematocrit, mean ± SD	0.27 ± 0.04
Postoperative serum creatinine (μmol/L), mean ± SD	90.68 ± 28.07
Serum creatinine at 3 months postoperatively (μmol/L), mean ± SD	85.55 ± 22.84
Total complications*, n (%)	
Grade I	2 (16.67)
Grade II	8 (66.67)
Grade III	3 (25.00)
Follow-up duration (months), mean ± SD	33.25 ± 17.03
Follow-up recurrence, n (%)	
Yes	0 (0.00)
No	12 (100.00)

*VAS, Visual Analogue Scale. Based on the Clavien–Dindo classification system, some patients experienced multiple complications simultaneously.

## 4 Discussion

The primary finding of this study is that 3D laparoscopy, when combined with membrane anatomy, can effectively treat ruptured RAML, particularly in terms of selecting the optimal timing for surgery. Although delayed surgery is associated with lower perioperative risks, our study demonstrates that single-stage 3D laparoscopic surgery achieves high renal preservation rates and reduces postoperative complications. This suggests that 3D laparoscopy not only provides high surgical precision but also plays a key role in preserving kidney function, offering a new perspective and treatment pathway for managing ruptured renal angiomyolipoma.

RAML is a common benign renal tumor composed primarily of blood vessels, smooth muscle, and adipose tissue. One of the main treatment goals is to preserve renal units to the maximum extent, especially in cases of ruptured bleeding, where renal preservation becomes a critical measure of surgical success (17). Historically, nephrectomy has been the standard treatment due to difficulties in controlling bleeding and the complex location of the tumor. However, with advances in surgical technology, kidney-preserving surgery has gradually become the preferred treatment for renal angiomyolipoma.

Our study demonstrates that the combination of 3D laparoscopy and membrane anatomy allows for precise identification of the blood vessels and key anatomical structures surrounding the renal angiomyolipoma. This approach effectively controls bleeding and maximizes the preservation of normal renal tissue (18). As a result, patients experience better postoperative recovery of renal function with minimal kidney damage, leading to significantly improved renal preservation rates. In cases of ruptured bleeding, this technique not only helps reduce intraoperative blood loss but also lowers the incidence of postoperative complications, offering significant advantages over traditional open surgery.

Although previous studies generally suggest that delayed surgery after ruptured renal angiomyolipoma may be safer—due to tumor and hematoma organization and reduced surrounding tissue inflammation—delayed surgery’s main advantage lies in the clearer boundaries between the tumor and surrounding tissues. This makes it easier to control bleeding and minimize damage to important blood vessels (15, 19). However, our findings suggest that despite the safety of delayed surgery, single-stage 3D laparoscopic surgery remains highly valuable. The 3D laparoscopy technology provides a stereoscopic view and high-definition imaging, making the kidney and surrounding anatomical structures clearer, thereby helping surgeons precisely identify and protect critical renal structures, especially when the hematoma has not yet fully organized after the rupture of renal angiomyolipoma.

Additionally, early postoperative treatment can alleviate symptoms rapidly, prevent further hematoma expansion after rupture, and reduce the occurrence of acute hemorrhagic shock. This approach is particularly important for critically ill patients, especially those with hematomas that have not fully organized but are still in an acute hemorrhagic state. In our study, although the surgical time was extended (average of 311.83 ± 85.46 minutes), the renal preservation rate was 100%, and no grade IV–V complications, perioperative death, conversion to radical nephrectomy, or renal unit loss occurred. Although three grade IIIa complications were observed, all were postoperative perirenal fluid collections or hematomas and were successfully managed by image-guided percutaneous drainage and supportive treatment. Nevertheless, the occurrence of grade IIIa complications also highlights the technical complexity of treating ruptured RAML and the need for careful postoperative monitoring. Renal function was effectively preserved, and no tumor recurrence was observed during follow-up. These results suggest that single-stage 3D laparoscopic surgery after hemorrhage stabilization may be a feasible treatment option for selected patients with ruptured RAML, with acceptable perioperative safety, nephron-sparing potential, and postoperative recovery.

One of the core advantages of 3D laparoscopy is its ability to provide a stereoscopic view, which enhances the surgeon’s understanding of the kidney and surrounding structures. When combined with membrane anatomy, 3D laparoscopy enables precise dissection of the kidney and surrounding blood vessels, renal fascia, and other critical structures, facilitating efficient vascular closure and reducing intraoperative bleeding (20, 21). The application of membrane anatomy further aids in identifying target vessels and determining the extent of the hematoma, providing accurate anatomical landmarks for treating ruptured renal angiomyolipoma. This anatomical framework allows the surgeon to avoid injury to critical blood vessels, thereby minimizing the risk of damage to the kidney and other organs (13). Furthermore, the design of instruments and trocar ports in 3D laparoscopy plays a crucial role in optimizing surgical efficiency. By strategically placing multiple trocar ports, the surgeon can operate multiple instruments simultaneously, which significantly enhances the efficiency and precision of the surgery. This is especially important for managing complex cases like ruptured renal angiomyolipoma, ensuring a smoother and more effective surgical process.

In the present study, 10 patients required intraoperative blood transfusion. Although this rate was relatively high and may appear to contrast with the concept of effective bleeding control, it should be interpreted in the emergency clinical context of ruptured RAML. Unlike elective treatment for unruptured RAML, patients with ruptured RAML often present with acute retroperitoneal hemorrhage, hematoma formation, decreased hemoglobin levels, and varying degrees of hemodynamic compromise before definitive surgical treatment. Therefore, intraoperative transfusion in this setting reflects not only newly occurring surgical blood loss but also the preoperative hemorrhagic burden, correction of pre-existing anemia, and the need to maintain perioperative hemodynamic stability. In a previous single-center series of emergency retroperitoneal laparoscopic partial nephrectomy for ruptured RAML, He et al. reported an intraoperative transfusion rate of 33.3% (5/15) (22). The higher transfusion rate in our cohort may be related to differences in baseline hemorrhagic severity, preoperative hemoglobin levels, hematoma volume, timing of intervention, and institutional transfusion thresholds. Accordingly, the present findings should be interpreted as indicating acceptable intraoperative bleeding control after hemorrhage stabilization rather than complete avoidance of transfusion.

AE plays a crucial role in the management of ruptured RAML. As a minimally invasive procedure, interventional therapy can rapidly control active bleeding, stabilize hemodynamic status, reduce tumor vascularity, and create favorable conditions for subsequent surgery. Previous studies have shown that interventional therapy may help minimize renal damage and reduce the need for more complex surgical interventions (9, 23). However, embolization also has limitations, as it may not provide definitive treatment for RAML, particularly in patients with multiple lesions or large aneurysms, in whom recurrence and reintervention risks may be higher (7, 24). In our treatment strategy, SAE was therefore used mainly for short-term hemorrhage stabilization and perioperative optimization rather than as the final curative treatment. Subsequent membrane anatomy-based 3D laparoscopic PN was performed to achieve definitive lesion removal and renal parenchymal preservation. Nevertheless, because all patients in this retrospective case series underwent preoperative arterial embolization, the respective contributions of embolization and the surgical technique cannot be quantitatively separated. From a clinical perspective, preoperative embolization likely contributed mainly to hemorrhage control, reduction of tumor vascularity, and stabilization of the operative field, whereas membrane anatomy-based 3D laparoscopic PN may have contributed mainly to precise anatomical dissection, lesion removal, and nephron preservation. Therefore, the observed perioperative outcomes should be interpreted as the combined effect of these two complementary components rather than the effect of either intervention alone.

Despite the potential feasibility of 3D laparoscopy combined with membrane anatomy in treating ruptured renal angiomyolipoma, several limitations should be acknowledged. First, this was a single-center retrospective descriptive case series with only 12 patients, which may introduce selection bias and limit the generalizability of the findings. Second, because no delayed surgery group, embolization-only group, open surgery group, or other nephron-sparing control group was included, we could not directly compare different treatment strategies or determine the independent contribution of preoperative AE versus the surgical technique to bleeding control, renal function preservation, postoperative recovery, recurrence, reintervention, or long-term outcomes. Third, the follow-up period was relatively short, limiting assessment of long-term renal function and tumor recurrence. Therefore, large-sample, multicenter prospective comparative studies, ideally using matched cohorts or propensity-score methods, are needed to further validate the safety, effectiveness, and potential clinical value of this treatment strategy.

## 5 Conclusion

Following preoperative arterial embolization, membrane anatomy-based 3D laparoscopic PN may be a feasible nephron-sparing option for selected patients with ruptured RAML. In this small single-center retrospective series, this approach was associated with acceptable perioperative safety, renal function preservation, and postoperative recovery. Further prospective comparative studies are required to confirm its safety, effectiveness, and potential clinical value.

## Data Availability

The raw data supporting the conclusions of this article will be made available by the authors, without undue reservation.
